# Comprehensive physiological, transcriptomic, and metabolomic analyses reveal the synergistic mechanism of *Bacillus pumilus* G5 combined with silicon alleviate oxidative stress in drought-stressed *Glycyrrhiza uralensis* Fisch.

**DOI:** 10.3389/fpls.2022.1033915

**Published:** 2022-12-08

**Authors:** Xin Ma, Zhanchao Xu, Duoyong Lang, Li Zhou, Wenjin Zhang, Xinhui Zhang

**Affiliations:** ^1^ College of Pharmacy, Ningxia Medical University, Yinchuan, China; ^2^ Laboratory Animal Center, Ningxia Medical University, Yinchuan, China; ^3^ Ningxia Engineering and Technology Research Center of Regional Characteristic Traditional Chinese Medicine, Ningxia Collaborative Innovation Center of Regional Characteristic Traditional Chinese Medicine, Key Laboratory of Ningxia Minority Medicine Modernization, Ministry of Education, Yinchuan, China

**Keywords:** drought stress, *Glycyrrhiza uralensis*, *Bacillus pumilus* G5 combined with silicon, AsA–GSH cycle, carotenoid biosynthesis, flavonoid biosynthesis

## Abstract

*Glycyrrhiza uralensis* Fisch. is often cultivated in arid, semi-arid, and salt-affected regions that suffer from drought stress, which leads to the accumulation of reactive oxygen species (ROS), thus causing oxidative stress. Plant growth-promoting bacteria (PGPB) and silicon (Si) have been widely reported to be beneficial in improving the tolerance of plants to drought stress by maintaining plant ROS homeostasis. Herein, combining physiological, transcriptomic, and metabolomic analyses, we investigated the response of the antioxidant system of *G. uralensis* seedlings under drought stress to *Bacillus pumilus* (G5) and/or Si treatment. The results showed that drought stress caused the overproduction of ROS, accompanied by the low efficiency of antioxidants [i.e., superoxide dismutase (SOD), catalase (CAT), peroxidase (POD), the ascorbate (AsA)–glutathione (GSH) pool, total carotenoids, and total flavonoids]. Inversely, supplementation with G5 and/or Si enhanced the antioxidant defense system in drought-stressed *G. uralensis* seedlings, and the complex regulation of the combination of G5 and Si differed from that of G5 or Si alone. The combination of G5 and Si enhanced the antioxidant enzyme system, accelerated the AsA–GSH cycle, and triggered the carotenoid and flavonoid metabolism, which acted in combination *via* different pathways to eliminate the excess ROS induced by drought stress, thereby alleviating oxidative stress. These findings provide new insights into the comparative and synergistic roles of PGPB and Si in the antioxidant system of plants exposed to drought and a guide for the application of PGPB combined with Si to modulate the tolerance of plants to stress.

## Introduction

Drought stress is a major environmental constraint that limits plant growth and development, crop productivity, and geographic distribution, especially in arid and semi-arid regions of the world (Carter and Dickman, 2022; Wu et al., 2022a). Drought limited the water uptake in plants and the nutrient absorption and transportation from the roots (Khan et al., 2020a), reduced the utilization rate and metabolism of nutrients, and increased the production of reactive oxygen species (ROS) (Jan et al., 2019; Rane et al., 2022). Excessive levels of ROS impair the enzyme activities, degrade proteins, and eventually induce plant cell death (Qi et al., 2018). Correspondingly, drought-exposed plants have evolved several defense mechanisms to maintain ROS homeostasis and protect cells against oxidative damage, and the antioxidant system serves as the first line of defense in eradicating the stress-induced excess ROS, which plays a key role in the response of plants to environmental stresses (Kartik et al., 2021). Thus, improving plant antioxidant pathways appears a useful strategy to increasing the tolerance of plants to drought stress.

In the antioxidant defense system, superoxide dismutase (SOD), catalase (CAT), and peroxidase (POD) are the major contributors to stress tolerance that mediate the protection of important physiological and biochemical processes (Hasanuzzaman et al., 2017). Simultaneously, the ascorbate (AsA)–glutathione (GSH) cycle is considered to control the levels of ROS through its scavenging activity and to contribute to redox sensing and signaling (Mittler, 2017). AsA and GSH, as powerful antioxidants of plant cells, can detoxify H_2_O_2_ to H_2_O, and the metabolic pools of AsA and GSH are tightly linked by the activity of the enzymes involved in the AsA–GSH cycle, i.e., ascorbate peroxidase (APX), glutathione peroxidase (GPX), monodehydroascorbate reductase (MDHAR), dehydroascorbate reductase (DHAR), and glutathione reductase (GR) (Mishra et al., 2022). Moreover, as the predominant antioxidants, carotenoids and flavonoids participate in the maintenance of redox buffering for the optimal performance of important pathways such as energy metabolism and photosynthetic electron transport (Ahanger and Agarwal, 2017; Das and Roychoudhury, 2014; Huchzermeyer et al., 2022). Thereby, each antioxidant plays a specific role in combating the specific type of ROS induced by stress.

Plant growth-promoting bacteria (PGPB), which alleviate drought stress, can produce exopolysaccharides and siderophores, facilitate the synthesis of 1-aminocyclopropane-1-carboxylate deaminase (ACCD), amplify nitrogen fixation and phosphate solubilization, improve the photosynthesis rate, regulate phytohormones and osmotic pressure, activate the antioxidant defense response system to scavenge ROS, modulate the transcription of the stress response genes, and induce the drought-tolerant change of host plants (Ilangumaran and Smith, 2017; Pathania et al., 2020; Kabiraj and Majhi, 2020; Zheng et al., 2022). Previous studies reported the bio-inoculation of PGPB to mitigate drought stress through the regulation of the antioxidant defense in a number of plants, including *Amaranthus tricolor*, *Glycyrrhiza uralensis* Fisch., *Zea mays*, and *Oryza sativa* L. (Gusain et al., 2015; Sarker and Oba, 2018a; Xie et al., 2019; Vishnupradeep et al., 2022). Similarly, Si is a widely recognized beneficial element in plants, and its mechanisms in strengthening drought tolerance, such as water balance, photosynthetic assimilation, gas exchange, nutrient absorption, secondary metabolite production, and antioxidant defense, have been reported (Zahedi et al., 2020; Sattar et al., 2020; Cui et al., 2021; Souri et al., 2021). Similar to PGPB, the protective effects of Si against oxidative damage have been documented in different plants, and the mechanism varies in different species (Khattab et al., 2014; Biju et al., 2017; Coskun et al., 2019; Cao et al., 2020). Recently, the synergistic effects of PGPB and Si for the amelioration of abiotic stress in plants have been examined. Fatemi et al. (2020) reported that the combined application of PGPB and Si produced better results in improving the growth traits of *Coriandrum sativum* under heavy metal stress compared to the single treatment. Furthermore, the combination of PGPB and Si conferred promising results in the mitigation of salinity stress in cucumber and mung bean (Mahmood et al., 2016; Mahmood et al., 2017; Kaloterakis et al., 2021). In wheat, the combined application of PGPB strains and Si improved drought tolerance by increasing the nutrient uptake and upregulating the antioxidant enzymes (SOD, POD, and CAT), and the effect of this combined treatment was better than that of the individual treatments (Akhtar et al., 2021).

*G. uralensis*, a perennial herb of the legume family, is commonly used as a food sweetener, tobacco flavoring agent, and Chinese medicine (Dang et al., 2021; Zhang et al., 2022; Bi et al., 2022). Due to its wide cultivation in arid and semi-arid desert steppes and edges, the yield of *G. uralensis* has been severely reduced (Li et al., 2022; Liu et al., 2022). Thus, it is of considerable economic and social importance to develop an effective method for improving the drought tolerance of cultivated *G. uralensis*. Although the specific role of PGPB or Si in the amelioration of drought stress has been well established in *G. uralensis* (Zhang et al., 2020; Zhang et al., 2021a), the comparative and synergistic application of PGPB and Si in alleviating drought stress in *G. uralensis* has not been thoroughly studied. Therefore, the objective of this study was to analyze the comparative and synergistic roles of PGPB and Si in the antioxidant system of *G. uralensis* under drought stress using physiological, transcriptomic, and metabolomic techniques, aiming to determine whether they trigger similar responses, use the same mechanisms, and whether they can be used together for synergistic benefits to improve drought tolerance in *G. uralensis*.

## Materials and methods

### Plant material, *B. pumilus* material, and preparation of *Bacillus* inoculate

*G. uralensis* seeds were collected from wild plants in Urad Front Banner, Inner Mongolia, China, in September 2019. Healthy seeds were selected and stored in a kraft paper bag at 4°C until use.

The G5 strain utilized in this study was obtained from *G. uralensis* roots and was identified as *B. pumilus* by Sangon Biotech Co., Ltd. (Shanghai, China). The strain was preserved at the China National Center for Total Culture of Microbes under preservation no. CGMCC 16879 and is listed in NCBI as MT 822869.

G5 was cultured at 28°C for 24 h in nutrient agar solid medium (3 g beef extract, 10 g tryptone, 5 g NaCl, 1,000 ml H_2_O, and 16 g agar pH 7.0–7.2) and then inoculated in sterilized nutrient broth liquid medium (3 g beef extract, 10 g tryptone, 5 g NaCl, and 1,000 ml H_2_O, pH 7.0–7.2) for 48 h with shaking at 180 rpm and 28°C. The optical density (OD_600_) was adjusted to about 1 with diluted sterile water, and the concentration of G5 was 10^8^ CFU/ml. As shown in **Supplementary Figure S1**, G5 and Si were used in combination according to the absence of a bacteriostatic band (Zheng et al., 2022).

### Plant growth conditions and treatments

The seeds of *G. uralensis* were steeped with concentrated sulfuric acid for 45 min to break the seed coat, surface sterilized with 0.1% H_2_O_2_ for 10 min, cleaned three times using distilled water, and finally imbibed in distilled water for 8 h at 25°C. Each pot was filled with 1,500 g of autoclaved and fully dried sandy soil collected from native desert regions and then pre-irrigated with 300 ml of distilled water. Subsequently, 65 water-absorbing fully filled seeds were selected and sown uniformly, then covered with 380 g of sandy soil in each pot. The experiment was conducted in an indoor environment with natural light and an air temperature of 23–28°CC during the day and night.

The experiment had a randomized design and included the following groups: і) control (CK) group, where plants were watered to keep 65%–75% of the saturation moisture content; ii) drought stress (D) group, in which plants were watered to keep 35%–45% of the saturation moisture content; iii) drought stress with G5 (D+G5) group, where plants were watered to keep 35%–45% of the saturation moisture content and G5 was added (10^8^ CFU/ml^-1^); iv) drought stress with Si (D+Si) group, where plants were watered to keep 35%–45% of the saturation moisture content and Si was added (2 mM); and v) drought stress with combined G5 and Si application (D+G5+Si) group, in which plants were watered to keep 35%–45% of the saturation moisture content and both G5 (10^8^ CFU/ml) and Si (2 mM) were applied. Si was added as 2 mM K_2_SiO_3_. The concentration of G5 inoculation was 10^8^ CFU/ml. A solution of the exogenous substance (G5, Si, or G5+Si) was prepared in 300 ml distilled water and irrigated the roots of *G. uralensis* seedlings after the appearance of the third true leaf. After 5 days, drought stress was simulated by controlling the saturated moisture content at 35%–45% (using the weighing method).

All treatments had three replications, and all pots were randomly arranged and periodically rotated to minimize the effect of environmental heterogeneity. The samples were collected 15 days after drought, immediately frozen in liquid nitrogen, and then stored at −80°CC until use.

### Measurement of the oxidative stress marker contents

Measurement of the production rate of $O_{2}^{\cdot -}$ was performed according to Siddiqui et al. (2020), with slight modifications. Absorbance was measured at 530 nm, and the standard curve was constructed with NaNO_2_. The H_2_O_2_ content was determined according to Siddiqui et al. (2020), with slight modifications. Absorbance was measured at 410 nm, and the standard curve was obtained using H_2_O_2_. The malondialdehyde (MDA) content was determined using the method of Zhou et al. (2004), with slight changes, and absorbance was measured at 532 and 600 nm.

### Measurement of the activities of SOD, CAT, and POD

The activity of SOD as determined using reagent kits (Beijing Solarbio Science & Technology Co., Ltd., Beijing, China) according to the manufacturer’s instructions. Absorbance was measured at 560 nm. A unit of SOD activity was defined as the quantity of enzyme required to reduce the inhibition of nitroblue tetrazolium by 50%. The activity of CAT was assessed according to Hasanuzzaman et al. (2011), with minimal changes. A unit of CAT activity was defined as a 0.01-unit change in absorbance per minute. The activity of POD was assessed using the method of Pan et al. (2006), with slight modifications. A unit of POD activity was defined as a 0.01-unit change in absorbance per minute.

### Measurement of the contents of key compounds related to the AsA–GSH cycle

The AsA content was determined according to the method of Hodges et al. (1997), with minor modifications. The standard curve was constructed with the AsA standard and the absorbance measured at 534 nm. The DHA content was estimated as the difference between the total AsA content and the AsA content, and absorbance was measured at 534 nm (Hodges et al., 1997).

The GSH and GSSG contents were determined using reagent kits (Beijing Solarbio Science & Technology Co., Ltd.) according to the manufacturer’s instructions. Absorbance was measured at 412 nm.

### Measurement of the activities of key enzymes related to the AsA–GSH cycle

The activity of APX as assayed according to the method of Lopez-Delacalle et al. (2021), with minor modifications. Absorbance was measured at 240 nm, and one unit of APX activity was defined as 0.01 unit of absorbance change per minute.

The activity of MDHAR was assayed as described previously (Hossain et al., 1984), with minor modifications. Absorbance was measured at 340 nm, and the activity was calculated based on the change in absorbance after 1 min (with an extinction coefficient of 6.2 mM^−1^ cm^−1^).

The activity of DHAR was measured as previously described (Nakano and Asada, 1981), with minor modifications. Absorbance was measured at 265 nm, and the activity was calculated based on the change in absorbance after 1 min (the extinction coefficient was 14 mM^−1^ cm^−1^).

The activity of GR was measured as previously described (Cakmak et al., 1993), with minor modifications. The change in absorbance was recorded at 340 nm within 1 min (with an extinction coefficient of 6.2 mM^−1^ cm^−1^).

The activity of GPX was determined using reagent kits (Beijing Solarbio Science & Technology Co., Ltd.) according to the manufacturer’s instructions. Absorbance was measured at 412 nm, and one unit of GPX activity was defined as the oxidation of 1 nmol GSH per minute.

### Measurement of the contents of total carotenoids and total flavonoids

The total carotenoid content was estimated by extracting the leaves using 96% ethanol following the method described by Lichtenthaler et al. (1987). Absorbance was read at wavelengths of 646, 663, and 470 nm.

The total flavonoid content was calculated according to the method of Zhang et al. (2019), with minor modifications. Absorbance was recorded at 530 nm, and the content was quantified to the standard curve of rutin.

### RNA extraction, cDNA library construction, and RNA-seq

Transcriptome sequencing was carried out by Beijing Baimeike Company. The experimental process followed the method provided by Oxford Nanopore Technologies, which included the following steps: 1) *G. uralensis* seedlings collected from the five treatment groups (CK, D, D+G5, D+Si, and D+G5+Si) were ground and biologically replicated for RNA preparation. Three biological replicates per sample were used for the RNA sequencing (RNA-seq) experiments; 2) RNA was extracted and its purity, concentration, and integrity were examined; 3) a library was constructed, which included primer annealing, reverse transcription into cDNA, and oligo switching, as well as synthesis of the complementary chains, DNA damage and terminal repair, and magnetic bead purification; and 4) finally, the sequencing connector was added for on-machine sequencing.

### Transcriptome data assembly

For data assembly, low-quality (length< 500 bp, *Q* score< 7) sequences and ribosomal RNA sequences from the original landing sequence were filtered and the full-length sequence according to the presence of primers at both ends of the sequence was obtained. The full-length sequence obtained in the previous step was polished to obtain a consistent sequence. Contig comparisons with reference genomes or constructed contig sequences were performed to remove redundancy.

### Transcription quantification

Transcriptome sequencing can be simulated as a random sampling process. To make the number of fragments truly reflect the expression levels of the transcripts, it is necessary to normalize the number of mapped reads in the sample. Counts per million (CPM) (Zhou et al., 2014) was used as an indicator to measure the expression levels of transcripts or genes.

### Quantification of the gene/transcript expression levels and differential expression analysis

Full-length reads were mapped to the reference transcriptome sequence. Reads with a match quality above 5 were further used for quantification. The expression levels were estimated as reads per gene/transcript per 10,000 mapped reads.

For samples with biological replicates, differential expression analysis of two conditions/groups was performed using the DESeq R package (1.18.0). DESeq provides statistical routines to determine the differential expression in digital gene expression data using a model based on the negative binomial distribution. The resulting *p*-values were adjusted using the Benjamini–Hochberg approach for controlling the false discovery rate. Genes with a *p*< 0.05 and fold change (FC) ≥ 1.5 found by DESeq were considered differentially expressed.

### Metabolite extraction and UPLC-MS/MS analysis

The LC/MS system used for metabolomics analysis was composed of the Waters Acquity I-Class PLUS ultra-high performance liquid tandem Waters Xevo G2-XS QTof high-resolution mass spectrometer. The column used was a Waters Acquity UPLC HSS T3 column (1.8 μm, 2.1 mm × 100 mm). For the positive ion mode, mobile phase A consisted of 0.1% formic acid aqueous solution, while mobile phase B was 0.1% formic acid acetonitrile. For the negative ion mode, mobile phase A was 0.1% formic acid aqueous solution, while mobile phase B consisted of 0.1% formic acid acetonitrile. The Waters Xevo G2-XS QTof high-resolution mass spectrometer can collect primary and secondary mass spectrometry data in MSe mode under the control of the acquisition software (MassLynx v4.2; Waters Corporation, Milford, MA, USA).

### Metabolite data preprocessing and annotation

The raw data collected using MassLynx v4.2 were processed with the Progenesis QI software for peak extraction, peak alignment, and other data processing operations based on the Progenesis QI software online METLIN database and the self-built library of Biomark for identification; at the same time, the theoretical fragment identification and mass deviation were all within 100 ppm.

### Metabolite data analysis

After normalizing the original peak area information with the total peak area, follow-up analysis was performed. Principal component analysis (PCA) and Spearman’s correlation analysis were used to examine the repeatability of the samples within the group and the quality control samples. The identified compounds were investigated for classification and pathway information in Kyoto Encyclopedia of Genes and Genomes (KEGG), Human Metabolome Database (HMDB), and the LIPID MAPS database. According to the grouping information, the difference multiples were calculated and compared, and Student’s *t*-test was used to calculate the significant difference *p*-value of each compound. The R language package “ropls” was used to perform orthogonal partial least squares discriminant analysis (OPLS-DA) modeling, with 200 times permutation tests performed to verify the reliability of the model. The variable importance in projection (VIP) value of the model was calculated using multiple cross-validation. The method of combining the difference multiple, the *p*-value, and the VIP value of the OPLS-DA model were used to screen the differential metabolites. The screening criteria were: FC ≥ 1, *p*< 0.05, and VIP > 1. Significance of the differential metabolites of the KEGG pathway enrichment was calculated using a hypergeometric distribution test.

### Statistical analyses

All treatments had three replications presented as the mean and the standard deviation of each experiment. One-way ANOVA was carried out and physiological data were examined for significant treatment differences using Duncan’s multiple range tests, and a *p*< 0.05 was considered as statistically significant. Correlation analysis of the physiological parameters in *G. uralensis* under the five conditions was conducted. PCA was performed on the physiological parameters of the response variables to separate the plants in the different treatments. The first two principal components, which accounted for the highest variation, were then used to plot two-dimensional scatter plots. All statistical analyses were performed using SPSS Statistics 25, Origin 2018 Statistics.

## Results

### Effects of G5 and/or Si on the oxidative stress markers in drought-stressed *G. uralensis* seedlings

**Figure 1:**
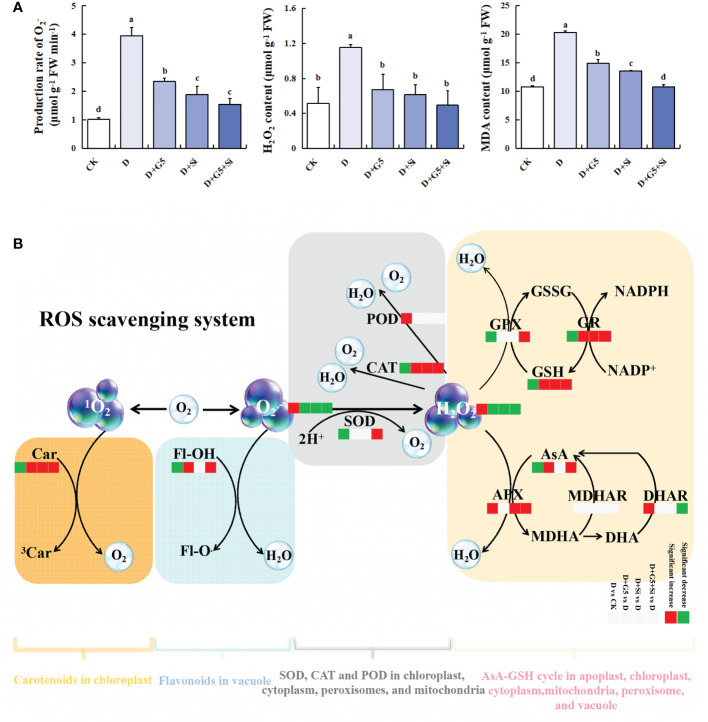
Effects of *Bacillus pumilus* (G5) and/or silicon (Si) on oxidative stress markers and the reactive oxygen species (ROS) scavenging system in drought-stressed *G lycyrrhiza uralensis* seedlings. **(A)** Oxidative stress markers. **(B)** ROS scavenging system. *CK*, control group; *D*, drought stress group; *D+G5*, drought stress with G5 group; *D+Si*, drought stress with Si group; *D+G5+Si*, drought stress with combined G5 and Si application group. *MDA*, malondialdehyde; *Car*, carotenoids; *Fl-OH*, *Fl-O*, flavonoids; *SOD*, superoxide dismutase; *POD*, peroxidase; *CAT*, catalase; *AsA*, ascorbate; *MDHA*, monodehydroascorbate; *DHA*, dehydroascorbate; *APX*, ascorbate peroxidase; *MDHAR*, monodehydroascorbate reductase; *DHAR*, dehydroascorbate reductase; *GSH*, glutathione; *GSSG*, glutathione disulfide; *GPX*, glutathione peroxidase; *GR*, glutathione reductase. *Different letters* within the different treatments indicate significant difference at *p*< 0.05. Values are the mean ± SE (*n* = 3).

As shown in **Figure 1A**, the production rate of $O_{2}^{\cdot -}$ and the contents of H_2_O_2_ and MDA were significantly increased in *G. uralensis* seedlings under drought stress (*p*< 0.01). Interestingly, G5, Si, and the combination of G5 and Si significantly decreased the $O_{2}^{\cdot -}$ production rate and the H_2_O_2_ and MDA contents in *G. uralensis* seedlings under drought stress (*p*< 0.05).

### Effects of G5 and/or Si on the activities of SOD, POD, and CAT in drought-stressed *G. uralensis* seedlings

**Figure 2:**
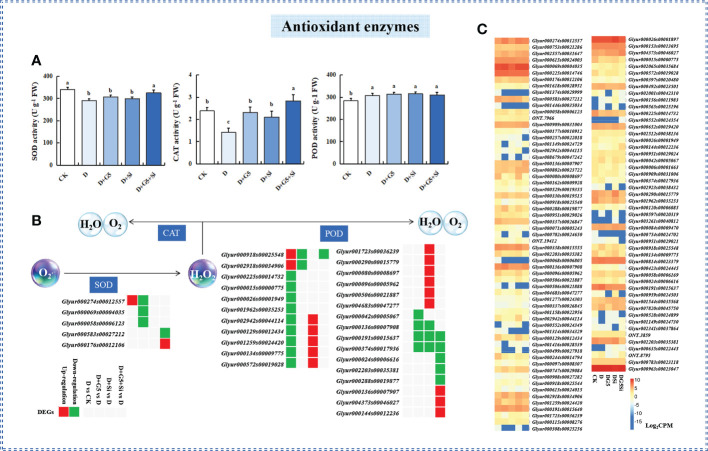
Effects of *Bacillus pumilus* (G5) and/or silicon (Si) on the antioxidant enzymes in drought-stressed *Glycyrrhiza uralensis* seedlings.**(A)** Activities of the antioxidant enzymes. **(B)** Expression patterns of the differentially expressed genes (DEGs) related to antioxidant enzymes. **(C)** Expression patterns of the genes related to antioxidant enzymes. *CK*, control group; *D*, drought stress group; *D+G5*, drought stress with G5 group; *D+Si*, drought stress with Si group; *D+G5+Si*, drought stress with combined G5 and Si application group. *SOD*, superoxide dismutase; *POD*, peroxidase; *CAT*, catalase. *Different letters* within the different treatments indicate significant difference at *p*< 0.05. Values are the mean ± SE (*n* = 3). Genes with *p*< 0.05 and fold change ≥ 1.5 were considered differentially expressed.

The activities of SOD and CAT were significantly decreased, while that of POD was significantly increased in *G. uralensis* seedlings under drought stress (*p*< 0.05). However, G5 or Si alone significantly increased only the activity of CAT, while their combination significantly increased the activities of both SOD and CAT in *G. uralensis* seedlings under drought stress (*p*< 0.05) (**Figures 1B**, **2A, B**).

The transcriptomic analysis showed that the genes encoding SOD and POD were differentially expressed under different treatments (*p*< 0.05, FC ≥ 1.5) (**Figures 2B, C**, **Table 1**). Specifically, the expression levels of one gene encoding SOD and two genes encoding POD were upregulated, while nine genes encoding POD were downregulated in *G. uralensis* seedlings under drought stress. Interestingly, G5 downregulated the expression of three genes encoding SOD and six genes encoding POD, while Si upregulated the expression of 11 genes encoding POD, but downregulated the expression of three genes encoding POD. On the other hand, the combination of G5 and Si upregulated the expression of one gene encoding SOD and three genes encoding POD, but downregulated the expression of one gene encoding SOD and six genes encoding POD in *G. uralensis* under drought stress.

**Table 1:** Regulation of the antioxidant enzyme-related differentially expressed genes (DEGs) in *Glycyrrhiza uralensis* Fisch. by *Bacillus pumilus* (G5) and/or silicon under drought stress.

Enzyme name	Definition (EC)	Gene name	Gene ID	Regulated			
				D *vs*. CK	D+G5 *vs*. D	D+Si *vs*. D	D+G5+Si *vs*. D
SOD	Superoxide dismutase [EC:1.15.1.1]	*SODCP*	Glyur000274s00012557	Up	Down	–	–
		*SOD1*	Glyur000069s00004035	–	Down	–	–
		*FSD3*	Glyur000058s00006123	–	Down	–	–
		*SODB*	Glyur000583s00027212	–	–	–	Down
		*At3g02290*	Glyur000176s00012106	–	–	–	Up
POD	Peroxidase [EC:1.11.1.7]	*GSVIVT00023967001*	Glyur000918s00025548	Up	Down	–	Down
		*PER64*	Glyur002918s00034906	Up	Down	–	–
		*PER52*	Glyur000225s00014732	Down	–	–	–
		*GSVIVT00037159001*	Glyur000015s00000775	Down	–	–	–
		*PER43*	Glyur000026s00001949	Down	–		–
		*PER12*	Glyur001962s00035253	Down	–		–
		*PER12*	Glyur002942s00044114	Down	–	Up	–
		*PER47*	Glyur000129s00012434	Down	–	Up	–
		*PER10*	Glyur001259s00024420	Down	–	Up	–
		*PER72*	Glyur000134s00009775	Down	–	Up	–
		*PER20*	Glyur000572s00019028	Down	–	Up	–
		*PNC1*	Glyur001723s00036239	–	–	Up	–
		*pod*	Glyur000290s00015779	–	–	Up	–
		*HRPN*	Glyur000080s00008697	–	–	Up	–
		*PER72*	Glyur000096s00005962	–	–	Up	–
		*PER16*	Glyur000506s00021887	–	–	Up	–
		*PER12*	Glyur004683s00047277	–	–	Up	–
		*PER50*	Glyur000042s00005067	–	Down	–	–
		*PNC2*	Glyur000136s00007908	–	Down	Down	–
		*PER12*	Glyur000191s00015637	–	Down	Down	Down
		*PER3*	Glyur000374s00017936	–	Down	Down	Down
		*PER53*	Glyur000024s00006616	–	–	–	Down
		*GSVIVT00023967001*	Glyur002203s00035381	–	–	–	Down
		*GSVIVT00023967001*	Glyur000288s00019877	–	–	–	Down
		*PNC2*	Glyur000136s00007907	–	–	–	Up
		*PER55*	Glyur004373s00046027	–	–	–	Up
		*GSVIVT00037159001*	Glyur000144s00012236	–	–	–	Up

CK, control group; D, drought stress group; D+G5, drought stress with G5 group; D+Si, drought stress with Si group; D+G5+Si, drought stress with combined G5 and Si application group.

### Effects of G5 and/or Si on the AsA–GSH cycle in drought-stressed *G. uralensis* seedlings

**Figure 3:**
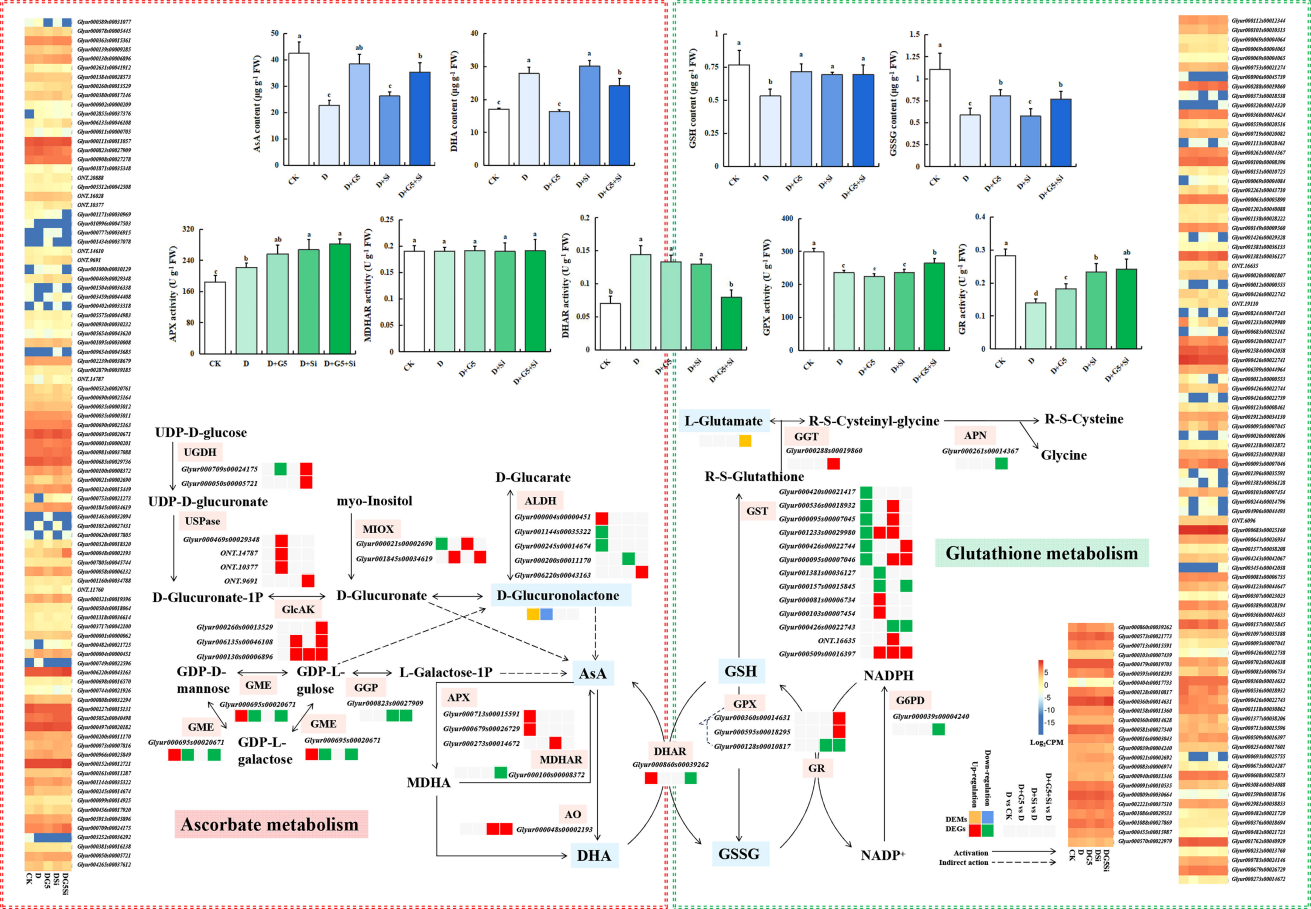
Effects of *Bacillus pumilus* (G5) and/or silicon (Si) on the ascorbate (AsA)–glutathione (GSH) cycle in drought-stressed *Glycyrrhiza uralensis* seedlings. *CK*, control group; *D*, drought stress group; *D+G5*, drought stress with G5 group; *D+Si*, drought stress with Si group; *D+G5+Si*, drought stress with combined G5 and Si application group. *AsA*, ascorbate; *MDHA*, monodehydroascorbate; *DHA*, dehydroascorbate; *GSH*, glutathione; *GSSG*, glutathione disulfide; *APX*, ascorbate peroxidase; *MDHAR*, monodehydroascorbate reductase; *DHAR*, dehydroascorbate reductase; *GPX*, glutathione peroxidase; *GR*, glutathione reductase; *UGDH*, UDP-glucose 6-dehydrogenase; *UDPase*, UDP-sugar pyrophosphorylase; *GlcAK*, glucuronokinase; *MIOX*, inositol oxygenase; *ALDH*, aldehyde dehydrogenase (NAD+); *GGP*, GDP-l-galactose phosphorylase; *GME*, GDP-mannose-3′,5′-epimerase; *AO*, l-ascorbate oxidase; *APX*, ascorbate peroxidase; *GPX*, glutathione peroxidase; *MDHAR*, monodehydroascorbate reductase; *DHAR*, dehydroascorbate reductase; *GST*, glutathione S-transferase; *G6PDH*, glucose-6-phosphate 1-dehydrogenase; *GGT*, gamma-glutamyl transpeptidase; *APN*, aminopeptidase N. *Different letters* within the different treatments indicate significant difference at *p*< 0.05. Values are the mean ± SE (*n* = 3). Genes with *p*< 0.05 and fold change ≥ 1.5 were considered differentially expressed; metabolites with *p*< 0.05, variable importance in projection (VIP) > 1, and fold change ≥ 1 were considered differentially expressed.

The contents of AsA, GSH, and GSSG were significantly decreased, but the DHA content was significantly increased in *G. uralensis* seedlings under drought stress (*p*< 0.05). Interestingly, G5 alone and the combination of G5 and Si significantly increased the contents of AsA, GSH, and GSSG, but decreased the DHA content, while Si only significantly increased the GSH content in *G. uralensis* seedlings under drought stress (*p*< 0.05) (**Figures 1B** and **3**). Moreover, according to the metabolomic analysis, drought stress increased the glucuronolactone content in *G. uralensis* seedlings (FC ≥ 1, *p*< 0.05, and VIP > 1). However, G5 decreased the glucuronolactone content, while the combination of G5 and Si increased the l-glutamate content in *G. uralensis* seedlings under drought stress (**Table 2**).

**Table 2:** Regulation of the ascorbate and glutathione cycle-related differentially expressed metabolites (DEMs) in *Glycyrrhiza uralensis* Fisch. by *Bacillus pumilus* (G5) and/or silicon under drought stress.

Pathway	Metabolite name	Metabolite ID	Regulated			
			D *vs*. CK	D+G5 *vs*. D	D+Si *vs*. D	D+G5+Si *vs*. D
Ascorbate and aldarate metabolism (ko00053)	Glucuronolactone	neg_1365	Up	Down	–	–
Glutathione metabolism (ko00480)	l-Glutamate	neg_927	–	–	–	Up

CK, control group; D, drought stress group; D+G5, drought stress with G5 group; D+Si, drought stress with Si group; D+G5+Si, drought stress with combined G5 and Si application group.

The transcriptomic analysis revealed that, for AsA metabolism, drought stress upregulated the expression of the genes encoding aldehyde dehydrogenase (ALDH) and GDP-mannose 3,5-epimerase (GME), but downregulated the expression of the genes encoding ALDH and inositol oxygenase (MIOX) in *G. uralensis* seedlings (*p*< 0.05, FC ≥ 1.5). However, G5 upregulated the expression of three genes encoding UDP-sugar pyrophosphorylase (UDPase), two genes encoding glucuronokinase (GlcAK), and one gene encoding MIOX, but downregulated the genes encoding UDP-glucose 6-dehydrogenase (UGDH) and GME in *G. uralensis* seedlings under drought stress. Si upregulated the expression of the genes encoding GlcAK and MIOX, but downregulated the expression of the genes encoding ALDH and GDP-l-galactose phosphorylase (GGP) in *G. uralensis* seedlings under drought stress. The combination of G5 and Si upregulated the expression of two genes encoding UGDH, the gene encoding UDPase, three genes encoding GlcAK, the gene encoding MIOX, and the gene encoding ALDH, but downregulated the expression of the genes encoding GME and GGP in *G. uralensis* seedlings under drought stress (*p*< 0.05, FC ≥ 1.5) (**Figure 3** and **Table 3**).

**Table 3:** Regulation of the ascorbate and glutathione cycle-related differentially expressed genes (DEGs) in *Glycyrrhiza uralensis* Fisch. by *Bacillus pumilus* (G5) and/or silicon under drought stress.

Enzyme name	Definition (EC)	Gene name	Gene ID	Regulated			
				D *vs*. CK	D+G5 *vs*. D	D+Si *vs*. D	D+G5+Si *vs*. D
UGDH	UDP-glucose 6-dehydrogenase [EC:1.1.1.22]	*UGD1*	Glyur000709s00024175	–	Down	–	Up
		*UGD1*	Glyur000050s00005721	–	–	–	Up
UDPase	UDP-sugar pyrophosphorylase [EC:2.7.7.64]	*RE1*	Glyur000469s00029348	–	Up	–	–
		*RE1*	ONT.14787	–	Up	–	–
		*RE1*	ONT.10377	–	Up	–	–
		*RE2*	ONT.9691	–	–	–	Up
GlcAK	Glucuronokinase [EC:2.7.1.43]	*SCRM*	Glyur000260s00013529	–	–	–	Up
		*BHLH35*	Glyur006135s00046108	–	Up		Up
		*GLCAK1*	Glyur000130s00006896	–	Up	Up	Up
MIOX	Inositol oxygenase [EC:1.13.99.1]	*MIOX1*	Glyur000021s00002690	Down	–	Up	–
		*MIOX2*	Glyur001845s00034619		Up	–	Up
ALDH	Aldehyde dehydrogenase (NAD^+^) [EC:1.2.1.3]	*ALDH3F1*	Glyur000004s00000451	Up	–	–	–
		*ALDH3H1*	Glyur001144s00035322	Down	–	–	–
		*ALDH3I1*	Glyur000200s00011170	–	–	Down	–
		*ALDH3H1*	Glyur000245s00014674	Down	–	–	–
		*ALDH2B7*	Glyur006220s00043163	–	–	–	Up
GME	GDP-d-mannose 3′, 5′-epimerase [EC:5.1.3.18]	*GME1*	Glyur000695s00020671	Up	Down	–	Down
GGP	GDP-l-galactose phosphorylase [EC:2.7.7.69]	*VTC2*	Glyur000823s00027909	–	–	Down	Down
**APX**	l-Ascorbate peroxidase [EC:1.11.1.11]	*PAP12*	Glyur000713s00015591	Up	–	–	–
		*CLEB3J9*	Glyur000679s00026729	Up	–	–	–
		*APX1*	Glyur000273s00014672	–	–	Up	–
AO	l-Ascorbate oxidase [EC:1.10.3.3]	–	Glyur000048s00002193	–	–	Up	Up
MDHAR	Monodehydroascorbate reductase (NADH) [EC:1.6.5.4]	*MDAR5*	Glyur000100s00008372	–	–	–	Down
DHAR	Glutathione dehydrogenase/transferase [EC:1.8.5.1 2.5.1.18]	*DHAR3*	Glyur000860s00039262	Up	–	–	Down
GPX	Glutathione peroxidase [EC:1.11.1.9]	*CSA*	Glyur000360s00014631	–	–	–	Up
		*GPX6*	Glyur000595s00018295	–	–	–	Up
		–	Glyur000128s00010817	–	–	Down	Down
G6PD	Glucose-6-phosphate 1-dehydrogenase [EC:1.1.1.49 1.1.1.363]	–	Glyur000039s00004240	–	–	–	Down
GST	Glutathione S-transferase [EC:2.5.1.18]	*HSP26-A*	Glyur000420s00021417	Down	–	–	–
		*PARB*	Glyur000536s00018932	Down	–	Up	–
		–	Glyur000095s00007045	Down	–	Up	–
		*PARA*	Glyur001233s00029980	Down	Up	Up	–
		*GST3*	Glyur000426s00022744	Down	–	–	Up
		–	Glyur000095s00007046	Down	–	Up	Up
		*HSP26*–*A*	Glyur001381s00036127	–	Down	–	–
		*GSTU17*	Glyur000157s00015845	–	Down	–	Down
		*GSTL3*	Glyur000081s00006734	–	Up	–	–
		–	Glyur000103s00007454	–	Up	–	–
		*PARC*	Glyur000426s00022743	–	–	Down	Down
		–	ONT.16635	–	–	Up	–
		–	Glyur000509s00016397	–	Up	Up	Up
GGT	Gamma-glutamyl transpeptidase/glutathione hydrolase [EC:2.3.2.2 3.4.19.13]	*GGT1*	Glyur000288s00019860	–	–	–	Up
APN	Aminopeptidase N [EC:3.4.11.2]	*At2g34460*	Glyur000261s00014367	–	–	–	Down

CK, control group; D, drought stress group; D+G5, drought stress with G5 group; D+Si, drought stress with Si group; D+G5+Si, drought stress with combined G5 and Si application group.

In GSH metabolism, drought stress also downregulated the expression of six genes encoding glutathione S-transferase (GST) in *G. uralensis* seedlings. G5 upregulated the expression of four genes, but downregulated the expression of two genes encoding GST; on the other hand, Si upregulated the expression of six genes encoding GST, but downregulated the expression of the gene encoding GST. The combination of G5 and Si upregulated the expression of three genes encoding GST and the gene encoding gamma-glutamyl transpeptidase (GGT), but downregulated the expression of the genes encoding GST and APN in *G. uralensis* seedlings under drought stress (*p*< 0.05, FC ≥ 1.5) (**Table 3**).

Furthermore, the activities of the AsA–GSH cycle enzymes (APX, GPX, DHAR, MDHAR, and GR) varied under the different treatments. Specifically, the activities of APX and DHAR were significantly increased, but those of GPX and GR were significantly decreased in *G. uralensis* seedlings under drought stress (*p*< 0.05). However, G5 significantly increased the activity of GR, while Si significantly increased the activities of APX and GR; on the other hand, the combination of G5 and Si significantly increased the activities of APX, GPX, and GR, but decreased the activity of DHAR in *G. uralensis* seedlings under drought stress (*p*< 0.05) (**Figures 1B** and **3**).

Moreover, transcriptomic analysis showed that drought stress upregulated the expression of the genes encoding APX and DHAR in *G. uralensis*. Si upregulated the expression of the genes encoding APX and l-ascorbate oxidase (AO), but downregulated the gene encoding GPX; however, the combination of G5 and Si upregulated the expression of the genes encoding AO and GPX, but downregulated the genes encoding MDHAR, DHAR, GPX, and glucose-6-phosphate 1-dehydrogenase (G6PD) in *G. uralensis* seedlings under drought stress (*p*< 0.05, FC ≥ 1.5) (**Table 3**).

### Effects of G5 and/or Si on carotenoid biosynthesis in drought-stressed *G. uralensis* seedlings

**Figure 4:**
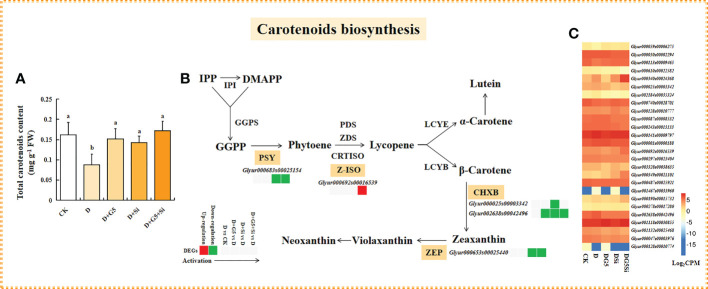
Effects of *Bacillus pumilus* (G5) and/or silicon (Si) on carotenoid biosynthesis in drought-stressed *Glycyrrhiza uralensis* seedlings. **(A)** Total carotenoid content. **(B)** Carotenoid biosynthesis process. **(C)** Expression patterns of the genes related to carotenoid biosynthesis. *CK*, control group; *D*, drought stress group; *D+G5*, drought stress with G5 group; *D+Si*, drought stress with Si group; *D+G5+Si*, drought stress with combined G5 and Si application group. *IPP*, isopentenyl diphosphate; *DMAPP*, dimethylallyl diphosphate; *GGPS*, geranylgeranyl diphosphate synthase; *PSY*, phytoene synthase; *PDS*, phytoene desaturase; *ZDS*, ξ-carotene desaturase; *CRTISO*, carotenoid isomerase; *Z-ISO*, ξ-carotene isomerase; *LCYE*, lycopene ϵ-cyclase; *LCYB*, lycopene β-cyclase; *CHXE*, ϵ-carotene hydroxylase; *CHXB*, β-carotene hydroxylase; *ZEP*, zeaxanthin epoxidase. *Different letters* within the different treatments indicate significant difference at *p*< 0.05. Values are the mean ± SE (*n* = 3). Genes with *p*< 0.05 and fold change ≥ 1.5 were considered differentially expressed.

As shown in **Figures 1B** and **4A**, the contents of total carotenoids were significantly decreased in *G. uralensis* seedlings under drought stress (*p*< 0.05). Interestingly, G5 and/or Si remarkably increased the total carotenoid content in *G. uralensis* under drought stress (*p*< 0.05).

The transcriptomic analysis revealed that G5 and Si individually downregulated the expression of the gene encoding β-carotene hydroxylase (CHXB), while Si alone and the combination of G5 and Si downregulated the expression of the genes encoding phytoene synthase (PSY), CHXB, and zeaxanthin epoxidase (ZEP) in *G. uralensis* seedlings under drought stress. Furthermore, the combination of G5 and Si upregulated the expression of the gene encoding ξ-carotene isomerase (Z-ISO) in *G. uralensis* under drought stress (*p*< 0.05, FC ≥ 1.5) (**Figures 4B, C** and **Table 4**).

**Table 4:** Regulation of the carotenoid biosynthesis-related differentially expressed genes (DEGs) in *Glycyrrhiza uralensis* Fisch. by *Bacillus pumilus* (G5) and/or silicon under drought stress.

Enzyme name	Definition (EC)	Gene name	Gene ID	Regulated			
				D *vs*. CK	D+G5 *vs*. D	D+Si *vs*. D	D+G5+Si *vs*. D
PSY	15-*cis*-Phytoene synthase [EC:2.5.1.32]	*PSY*	Glyur000683s00025154	–	–	Down	Down
Z-ISO	Zeta-carotene isomerase [EC:5.2.1.12]	*Z-ISO*	Glyur000692s00016539	–	–	–	Up
CHXB	β-carotene hydroxylase [EC:1.14.15.24]	*CA2*	Glyur000025s00003342	–	–	Down	–
		*CA2*	Glyur002638s00042496	–	Down	Down	Down
ZEP	Zeaxanthin epoxidase [EC:1.14.15.21]	–	Glyur000653s00025440	–	–	Down	Down

CK, control group; D, drought stress group; D+G5, drought stress with G5 group; D+Si, drought stress with Si group; D+G5+Si, drought stress with combined G5 and Si application group.

### Effects of G5 and/or Si on flavonoid biosynthesis in drought-stressed *G. uralensis* seedlings

**Figure 5:**
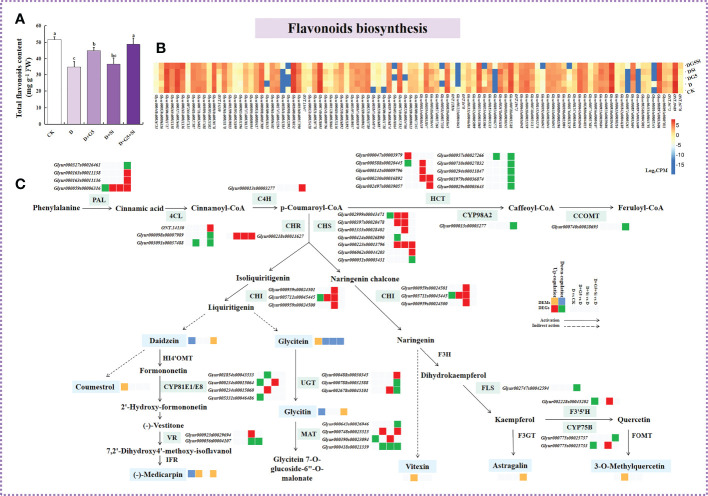
Effects of *Bacillus pumilus* (G5) and/or silicon (Si) on flavonoid biosynthesis in drought-stressed *Glycyrrhiza uralensis* seedlings. **(A)** Total flavonoid content. **(B)** Flavonoid biosynthesis pathway. **(C)** Expression patterns of the genes related to flavonoid biosynthesis. *CK*, control group; *D*, drought stress group; *D+G5*, drought stress with G5 group; *D+Si*, drought stress with Si group; *D+G5+Si*, drought stress with combined G5 and Si application group. *PAL*, phenylalanine ammonia-lyase; *4CL*, 4-coumarate-CoA ligase; *C4H*, *trans*-cinnamate 4-monooxygenase; *HCT*, shikimate *O*-hydroxycinnamoyltransferase; CYP98A2, 5-*O*-(4-coumaroyl)-d-quinate 3′-monooxygenase; *CCOMT*, caffeic acid 3-*O*-methyltransferase/acetylserotonin *O*-methyltransferase; *CHS*, chalcone synthase; *CHI*, chalcone isomerase; *CHR*, chalcone reductase; *HI4′OMT*, 2,7,4′-trihydroxyisoflavanone 4′-*O*-methyltransferase; *CYP81E1/E8*, isoflavone/4′-methoxyisoflavone 2′-hydroxylase; *VR*, vestitone reductase; *IFR*, 2′-hydroxyisoflavone reductase; *UGT*, isoflavone 7-*O*-glucosyltransferase; *MAT*, isoflavone 7-*O*-glucoside-6"-*O*-malonyltransferase; *F3H*, flavanone 3-hydroxylase; *FLS*, flavonol synthase; *F3′*,*5′H*, flavonoid 3′,5′-hydroxylase; *CYP75B*, flavonoid 3′-monooxygenase; *F3GT*, flavonol 3-*O*-glucosyltransferase. *Different letters* within the different treatments indicate significant difference at *p*< 0.05. Values are the mean ± SE (*n* = 3). Genes with *p*< 0.05 and fold change ≥ 1.5 were considered differentially expressed; metabolites with *p*< 0.05, and variable importance in projection (VIP) > 1, and fold change ≥ 1 were considered differentially expressed.

**Figures 1B** and **5A** display the contents of total flavonoids, which were significantly decreased in *G. uralensis* seedlings under drought stress (*p<* 0.05). However, G5 alone and the combination of G5 and Si remarkably increased the total flavonoid content in *G. uralensis* seedlings under drought stress (*p*< 0.05).

Moreover, the transcriptomic analysis revealed that drought stress induced the downregulation of the expression of the genes encoding phenylalanine ammonia lyase (PAL), shikimate *O*-hydroxycinnamoyltransferase (HCT), chalcone synthase (CHS), chalcone isomerase (CHI), isoflavone 7-*O*-glucoside 6"-*O*-malonyltransferase (MAT), flavonoid 3′,5′-hydroxylase (F3′,5′H), isoflavone/4′-methoxyisoflavone 2′-hydroxylase (CYP81E1/E8), and flavonoid 3′-monooxygenase (CYP75B), while it upregulated the expression of the gene encoding HCT in *G. uralensis* (*p*< 0.05, FC ≥ 1.5) (**Figures 5B, C** and **Table 5**).

**Table 5:** Regulation of the flavonoid biosynthesis-related differentially expressed genes (DEGs) in *Glycyrrhiza uralensis* Fisch. by *Bacillus pumilus* (G5) and/or silicon under drought stress.

Enzyme name	Definition (EC)	Gene name	Gene ID	Regulated			
				D *vs*. CK	D+G5 *vs*. D	D+Si *vs*. D	D+G5+Si *vs*. D
PAL	Phenylalanine ammonia-lyase [EC:4.3.1.24]	*PAL1*	Glyur000327s00026461	–	–	–	Down
		*PAL1*	Glyur000163s00011138	–	–	–	Up
		*PAL1*	Glyur000163s00011136	–	–	–	Up
		–	Glyur000059s00006316	Down	Up	Up	Up
4CL	4-Coumarate-CoA ligase [EC:6.2.1.12]	*4CLL7*	ONT.14130	–	–	–	Up
		*4CL1*	Glyur000098s00007989	–	–	–	Down
		*4CLL6*	Glyur003091s00037488	–	Down	–	Down
C4H	*trans*-Cinnamate 4-monooxygenase [EC:1.14.14.91]	–	Glyur000002s00000296	–	–	–	Up
HCT	Shikimate *O*-hydroxycinnamoyltransferase [EC:2.3.1.133]	–	Glyur000047s00003979	Up	–	–	–
		*AT1*	Glyur000588s00020445	Down	–	Up	–
		–	Glyur000141s00009796	–	–	Up	–
		*SHT*	Glyur000230s00016892	–	–	Up	Up
		*CER2*	Glyur002497s00039057	–	–	–	Up
		*SAT*	Glyur000957s00027266	–	Down	–	Down
		*HST*	Glyur000710s00027832	–	–	–	Down
		*CER26L*	Glyur000294s00011847	–	–	–	Down
		*PHT1*	Glyur001979s00036874	–	–	–	Down
		*SHT*	Glyur000029s00003643	–	–	–	Down
CYP98A2	5-*O*-(4-coumaroyl)-d-quinate 3′-monooxygenase [EC:1.14.14.96]	*CYP98A2*	Glyur000013s00003277	–	–	–	Down
CCOMT	Caffeoyl-CoA *O*-methyltransferase [EC:2.1.1.104]	*CCOMT*	Glyur000740s00028693	–	–	–	Down
CHS	Chalcone synthase [EC:2.3.1.74]	*CHS2*	Glyur000424s00026890	–	Down	–	–
		*CHS*	Glyur002999s00043471	Down	Up	Up	–
		*CHS6*	Glyur000397s00020478	–	Up	Up	–
		*CHS5*	Glyur001333s00028402	–	–	Up	–
		*CHS1*	Glyur000223s00013796	–	Up	Up	Up
		*CHS1*	Glyur006062s00044203	–	–	–	Up
		*CHS1*	Glyur000051s00003431	–	–	–	Down
CHR	Chalcone reductase	–	Glyur000218s00011627	–	Up	Up	Up
CHI	Chalcone isomerase [EC:5.5.1.6]	*CHI1B1*	Glyur000959s00024501	–	–	Up	–
		*CHI1*	Glyur005711s00045445	Down	Up	Up	–
		*CHI1B1*	Glyur000959s00024500	–	–	Up	–
CYP81E1/E8	Isoflavone/4′-methoxyisoflavone 2’-hydroxylase [EC:1.14.14.90 1.14.14.89]	*CYP81E1*	Glyur000234s00015064	Down	–	Up	–
		*CYP81E8*	Glyur005331s00046486	Down	–	–	–
		*CYP81E1*	Glyur000234s00015060	–	Up	–	–
		*CYP81E8*	Glyur001854s00043533	–	Down	–	–
VR	Vestitone reductase [EC:1.1.1.348]	–	Glyur000923s00029694	–	–	Up	–
		–	Glyur000056s00004107	–	–	Down	Down
UGT	Isoflavone 7-*O*-glucosyltransferase [EC:2.4.1.170]	*UGT13*	Glyur000488s00030345	–	–	–	Up
		*UGT1*	Glyur000788s00032588	–	–	–	Down
		*UGT2*	Glyur002678s00045101	–	–	Up	Down
MAT	Isoflavone 7-*O*-glucoside-6′-*O*-malonyltransferase [EC:2.3.1.115]	*PMAT1*	Glyur000643s00026946	–	–	–	Down
		*PMAT1*	Glyur000748s00025515	–	Up	–	Up
		*PMAT1*	Glyur000390s00023094	Down	–	Up	–
		*5MAT*	Glyur000418s00021339	–	Down	Down	Down
FLS	Flavonol synthase [EC:1.14.20.6]	*FLS*	Glyur002747s00042594	–	–	–	Down
F3′,5′H	Flavonoid 3′,5′-hydroxylase [EC:1.14.14.81]	*CYP75A1*	Glyur002228s00045202	Down	–	Up	–
CYP75B	Flavonoid 3′-monooxygenase [EC:1.14.14.82]	*CYP75B2*	Glyur000775s00025737	–	–	–	Down
		*CYP75B1*	Glyur000775s00025733	Down	–	Up	–

CK, control group; D, drought stress group; D+G5, drought stress with G5 group; D+Si, drought stress with Si group; D+G5+Si, drought stress with combined G5 and Si application group.

However, with the application of G5, the genes encoding PAL, CHS, CHR, CHI, CYP81E1/E8, and MAT were upregulated, but the genes encoding 4-coumarate-CoA ligase (4CL), CHS, CYP81E1/E8, MAT, and HCT were downregulated in *G. uralensis* under drought stress. With the application of Si, the genes encoding PAL, HCT, CHS, CHR, CHI, MAT, isoflavone 7-*O*-glucosyltransferase (UGT), vestitone reductase (VR), F3′,5′H, and CYP75B were upregulated, while those encoding MAT and VR were downregulated in *G. uralensis* under drought stress. Moreover, the combination of G5 and Si upregulated the genes encoding PAL, 4CL, *trans*-cinnamate 4-monooxygenase (C4H), HCT, CHS, CHR, MAT, and UGT, but downregulated the genes encoding PAL, 4CL, HCT, 5-*O*-(4-coumaroyl)-d-quinate 3′-monooxygenase (CYP98A2), caffeic acid 3-*O*-methyltransferase/acetylserotonin *O*-methyltransferase (CCOMT), CHS, VR, MAT, UGT, flavonol synthase (FLS), and CYP75B in *G. uralensis* under drought stress (*P*< 0.05 and FC ≥ 1.5) (**Figures 5B, C** and **Table 5**).

Moreover, metabolomic analysis showed that drought stress decreased the contents of daidzein, glycitin, and (−)-medicarpin, but increased the coumestrol and glycitein contents in *G. uralensis.* However, G5 increased the contents of (−)-medicarpin, vitexin, and 3-*O*-methylquercetin; similarly, the combination of G5 and Si increased the contents of daidzein, (−)-medicarpin, glycitin, and astragalin, while G5 and/or Si decreased the content of glycitein in *G. uralensis* under drought stress (*p*< 0.05, FC ≥ 1, VIP > 1) (**Figures 5B, C** and **Table 6**).

**Table 6:** Regulation of the flavonoid biosynthesis-related differentially expressed metabolites (DEMs) in *Glycyrrhiza uralensis* Fisch. by *Bacillus pumilus* (G5) and/or silicon under drought stress.

Metabolite name	Metabolite ID	Regulated			
		D *vs*. CK	D+G5 *vs*. D	D+Si *vs*. D	D+G5+Si *vs*. D
Glycitein	neg_10761	Up	Down	Down	Down
Vitexin	neg_5513	–	Up	–	–
Astragalin	neg_6043	–	–	–	Up
Coumestrol	neg_8315	Up	–	–	–
Daidzein	pos_13283	Down	–	–	Up
(−)-Medicarpin	pos_11757	Down	Up	–	Up
3-*O*-Methylquercetin	pos_8398	–	Up	–	–
Glycitin	neg_4482	Down	–	–	Up

CK, control group; D, drought stress group; D+G5, drought stress with G5 group; D+Si, drought stress with Si group; D+G5+Si, drought stress with combined G5 and Si application group.

### Hierarchical clustering with heatmap and principal component analysis

**Figure 6:**
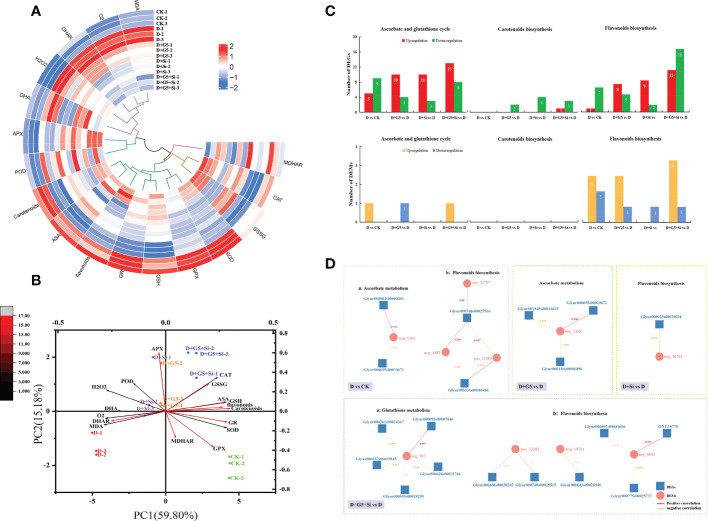
Hierarchical clustering with heatmap **(A)** and principal component analysis of the reactive oxygen species (ROS) scavenging system **(B)**, the number of differentially expressed genes (DEGs) and differentially expressed metabolites (DEMs) **(C)**, and correlation network map of the DEGs and DEMs involved in antioxidant biosynthesis **(D)** in drought-stressed *Glycyrrhiza uralensis* seedlings. *CK*, control group; *D*, drought stress group; *D+G5*, drought stress with G5 group; *D+Si*, drought stress with Si group; *D+G5+Si*, drought stress with combined G5 and Si application group. *MDA*, malondialdehyde; *AsA*, ascorbate; *MDHA*, monodehydroascorbic acid; *DHA*, dehydroascorbate; *GSH*, glutathione; *GSSG*, glutathione disulfide; *SOD*, superoxide dismutase; *POD*, peroxidase; *CAT*, catalase; *APX*, ascorbate peroxidase; *GPX*, glutathione peroxidase; *MDHAR*, monodehydroascorbate reductase; *DHAR*, dehydroascorbate reductase; *neg_1365*, glucuronolactone; *pos_11757*, (−)-medicarpin; *neg_4482*, glycitin; *pos_13283*, daidzein; *neg_10761*, glycitein; *neg_6043*, astragalin. **(A)** Values of the various parameters obtained in this study normalized and analyzed in the hierarchical clustering and heatmap. Two distinct clusters were identified at the variable level. In the heatmap, the *color scale* displays the intensity of the normalized values. **(B)** Principal component analysis. The *depth of the color of the arrow line* represents the degree of contribution, while the *angle of the arrow line* represents correlations. **(C)** In the number of differentially expressed genes (DEGs) and differentially expressed metabolites (DEMs), genes with *p*< 0.05 and fold change ≥ 1.5 were considered differentially expressed; metabolites with *p*< 0.05, variable importance in projection (VIP) > 1, and fold change ≥ 1 were considered differentially expressed. **(D)** Correlation network map showing the DEGs positively or negatively correlated with the DEMs, representing metabolite changes that may be positively or negatively regulated by the genes. Values on the DEG and DEM line represent the correlation coefficients.

The results of all the physiological–biochemical parameters (the oxidative stress markers, key substances, the enzymes related to the AsA–GSH cycle, and the contents of total carotenoids and total flavonoids) were subjected to heatmap analysis with hierarchical clustering (**Figure 6A**). Hierarchical clustering revealed the categorization of all the studied variables into two clusters: cluster 1, which covered MDA, $O_{2}^{\cdot -}$ , DHAR, H_2_O_2_, and the DHA variables, and cluster 2, which included total carotenoids, AsA, total flavonoids, GR, GSH, GPX, and the SOD variables. Compared with the D group, the variables of cluster 1 showed a decreasing pattern in most of the cases under the D+G5, D+Si, and D+G5+Si treatments, while these variables displayed an increasing trend in the D-treated compared to CK-treated *G. uralensis*. However, this effect was partially reversed in cluster 2; specifically, compared with the D group, the variables of cluster 2 showed an increasing pattern in some of the cases under the D+G5, D+Si, and D+G5+Si treatments, while these variables displayed a decreasing trend in most of the cases in the D-treated compared to CK-treated *G. uralensis*.

The results of the PCA related to all the physiological–biochemical parameters showed that PC1 explained 59.80% of the total variation, while PC2 accounted for 15.18% of the total variation (**Figure 6B**). PC1 formation covered the oxidative stress markers ( $O_{2}^{\cdot -}$ and MDA), non-enzymatic antioxidants (GSH, DHA, AsA, total flavonoids, and total carotenoids), and the enzymatic antioxidant (DHAR) and was separated by these parameters between plants in the CK and D treatment groups and between plants in the D+G5+Si and D+G5 treatment groups or the D+Si treatment. PC2 distinctly separated the plants given treatment D from those given the G5 and/or Si treatments because of the oxidative stress marker (H_2_O_2_), non-enzymatic antioxidant (GSSG), and the enzymatic antioxidants (APX, GPX, POD, CAT, GR, SOD, and MDHAR). Specifically, compared with the single treatment of G5 or Si, the combined G5 and Si treatment had a stronger correlation with the majority of the antioxidants (i.e., CAT, GSSG, ASA, GSH, total flavonoids, and total carotenoids) under drought stress, suggesting that the effects of the combination of G5 and Si on the antioxidants in *G. uralensis* under drought stress are greater than that of G5 or Si alone (**Figure 6B**).

### The number and correlation network map of the DEGs and DEMs involved in the biosynthesis of the major antioxidants of drought-stressed *G. uralensis* seedlings

As shown in **Figure 6C**, comparison of the D and CK treatments showed 14 differentially expressed genes (DEGs) and one differentially expressed metabolite (DEM) related to the AsA–GSH cycle and 10 DEGs and five DEMs related to flavonoid biosynthesis. These results indicated that drought mainly affects the ability of *G. uralensis* to scavenge ROS mainly by affecting the AsA–GSH cycle and flavonoid biosynthesis.

Interestingly, comparison of the D+G5 and D treatments, revealed 14 DEGs and one DEM related to the AsA–GSH cycle, two DEGs related to carotenoid biosynthesis, and eight DEGs and four DEMs related to flavonoid biosynthesis. The comparison of D+Si and D treatments revealed 13 DEGs related to the AsA–GSH cycle, four DEGs related to carotenoid biosynthesis, and 11 DEGs and one DEM related to flavonoid biosynthesis. In the D+G5+Si *vs*. D treatment, there were 21 DEGs and one DEM related to the AsA–GSH cycle, four DEGs related to carotenoid biosynthesis, and 30 DEGs and five DEMs related to flavonoid biosynthesis, From these results, it was observed that the numbers of DEGs and DEMs regulated by the combination of G5 and Si were the highest and that their regulatory effects were more diverse (**Figure 6C**).

To examine the relationship between the DEGs and DEMs in the G5- and/or Si-treated *G. uralensis* plants during drought stress, a co-expression network analysis of the DEGs and DEMs was conducted (**Figure 6D**). The co-expression networks of the DEGs and DEMs in the comparison between the D and CK treatments were found to be related to AsA metabolism and flavonoid biosynthesis. Interestingly, the co-expression networks of the DEGs and DEMs in the D+G5 *vs*. D treatment were only enriched in AsA metabolism, those in D+Si *vs*. D were only enriched in flavonoid biosynthesis, and the networks in D+G5+Si *vs*. D were mainly enriched in GSH metabolism and flavonoid biosynthesis. The results indicate that G5 and/or Si could modulate the co-expression of the DEGs and DEMs related to the biosynthesis of the major antioxidants in *G. uralensis* during drought stress, and the expression patterns were different depending on the application of G5 and/or Si. Notably, G5 combined with Si uniquely regulated the co-expression of the DEGs and DEMs related to GSH metabolism in *G. uralensis* under drought stress.

## Discussion

### G5 and/or Si reduced the oxidative stress markers in drought-stressed *G. uralensis* seedlings

Drought stress induced oxidative stress in plants caused by excessive ROS such as $O_{2}^{\cdot -}$ , ^1^O_2,_ and H_2_O_2_ (Choudhury et al., 2017; Laxa et al., 2019), which impaired the membrane lipids, proteins, and nucleic acids of plant cells and then correspondingly increased the level of MDA. Researchers have found that drought stress increased the contents of $O_{2}^{\cdot -}$ and H_2_O_2_ in *G. uralensis* (Zhang et al., 2017), *Z. mays* L. (Li et al., 2021b), and *Glycine max* (Chen et al., 2021) and increased the concentrations of MDA in *Triticum aestivum* L. (Nasirzadeh et al., 2021) and *Pimpinella anisum* L. (Esim et al., 2021). Similarly, in the present study, the $O_{2}^{\cdot -}$ and H_2_O_2_ production and the MDA concentration were significantly increased under drought stress in *G. uralensis*, indicating an oxidative burst and prompting cellular damage in plants due to water deficit (**Figure 1A**). Inversely, the production of $O_{2}^{\cdot -}$ and H_2_O_2_ was significantly reduced by G5 and/or Si, which could alleviate oxidative damage, and a decline in the MDA level was then correspondingly observed. These results are similar to the effects of Si on *Solanum lycopersicum* L. (Shi et al., 2016) and *T. aestivum* L. (Ma et al., 2016) and of PGPB on *Lolium perenne* L. (He et al., 2021). It is worth noting that, compared to single treatment with G5 or Si, alleviation of the effects of ROS accumulation in *G. uralensis* seedlings was greater when G5 and Si were combined; the levels of MDA were evenly comparable to those of the control treatment, indicating that both G5 and Si have synergistic effects on the mitigation of oxidative stress in *G. uralensis* seedlings.

### G5 and/or Si regulated the antioxidant enzyme system in drought-stressed *G. uralensis* seedlings

SOD, POD, and CAT are the main enzymes that scavenge ROS, and these antioxidant enzymes are found in various locations throughout plant cells and collaborate to detoxify ROS (Laxa et al., 2019). Drought stress altered the contents and the activities of the enzymes involved in ROS scavenging in different species (Ritonga et al., 2021; Meng et al., 2021). The present study showed that drought stress significantly decreased the activities of SOD and CAT in *G. uralensis*, indicating that drought stress remarkably inhibited the activity of some antioxidant enzymes; thus, the ROS generated by oxidative stress was not eradicated in time and led to its accumulation, resulting in cell damage. However, the activity of POD significantly increased under drought stress in *G. uralensis*; the reason behind this could be that the reduction in CAT activity was compensated by the increase in PODs, which can be used to scavenge the H_2_O_2_ under drought stress (Sarker and Oba, 2018a). Moreover, the transcriptomic analysis showed that drought stress upregulated one gene encoding SOD, which was not consistent with the trend of the enzyme activity, indicating that the gene might not be a key gene for the regulation of SOD activity in *G. uralensis* under drought stress (**Figure 2B**).

PGPB and/or Si play an important role in regulating the activities of antioxidant enzymes to mitigate the oxidative damage caused by drought stress (Rahimzadeh and Pirzad, 2017; Bukhari et al., 2021; Akhtar et al., 2021). Our research found that Si or G5 alone significantly increased the activity of CAT and that G5 combined with Si significantly increased the activities of SOD and CAT in *G. uralensis* seedlings under drought stress (**Figure 2A**), suggesting that the combination of G5 and Si had a unique regulatory effect that is different from the simple superposition of the effects of G5 and Si separately. Specifically, the combination of G5 and Si accelerated the dismutation of $O_{2}^{\cdot -}$ into H_2_O_2_ by improving the SOD activity in *G. uralensis* seedlings under the drought condition; subsequently, the excess H_2_O_2_ can be directly decomposed by boosting the activity of CAT, thus could be maintained as free radicals at a lower level and finally alleviate lipid peroxidation damage of the cell membrane in *G. uralensis* seedlings under drought stress. Complementarily, the transcriptomic analysis showed that the combination of G5 and Si upregulated the expression of one gene encoding SOD and three genes encoding POD, while it downregulated the expression of one gene encoding SOD and six genes encoding POD in *G. uralensis* under drought stress, implying that the effects of G5 and/or Si on the SOD and POD activities at physiological levels may be the result of the integrated action of all genes and related to the function of different genes encoding the same enzyme.

### G5 and/or Si accelerated the AsA–GSH cycle in drought-stressed *G. uralensis* seedlings

In addition to the antioxidant enzymes, the non-enzymatic components may also directly scavenge ROS. The AsA–GSH cycle is composed of AsA, GSH, and NADPH, which are continuously oxidized and reduced under the catalysis of several enzymes and maintain the integrity of the photosynthetic membranes under oxidative stress (Sarker and Oba, 2018b). Increases in the levels of GSH and AsA and the enzyme activities related to their biosynthesis and metabolism are strongly connected to the responses of plants to drought stress (Asthir et al., 2020).

In AsA metabolism, AsA synthesis is mainly affected by d-glucuronate, d-glucuronolactone, and l-galactose-1P. d-Glucuronateon the other hand, is synthesized from UDP-glucose or myoinositol. UDP-glucose can produce d-glucuronate through UDPase, UGDH, and GlcAK (Xiao et al., 2017), in which UDPase and UGDH catalyze UDP-glucose to the glucoronate-1P that was transformed to d-glucuronate by GlcAK, while myoinositol can produce to d-glucuronate with MIOX (Nepal et al., 2019). d-Glucuronolactone is converted from d-glucuronate or GDP-l-glucose. Moreover, d-glucuronolactone is generated by d-glucoronate under the catalysis of ALDH (Watanabe et al., 2007). Our results showed that drought stress increased the d-glucuronolactone content in *G. uralensis*, which might be due to the upregulation of the genes encoding GME and ALDH (**Figure 3**). Interestingly, the combination of G5 and Si upregulated the genes encoding UDPase, UGDH, and ALDH and three genes encoding GlcAK in *G. uralensis* under drought stress, exhibiting that this treatment combination might relieve the AsA deficiency by promoting d-glucuronate accumulation in *G. uralensis* under drought stress. For the synthesis of l-galactose-1P, GME catalyzed the conversion of GDP-d-mannose to GDP-l-galactose in the d-mannose/l-galactose pathway (Ma et al., 2014a). GGP is known to catalyze GDP-l-galactose to l-galactose-1-P (Dowdle et al., 2007). In this study, drought stress upregulated the gene encoding GME, while the combination of G5 and Si downregulated the genes encoding GME and GGP in *G. uralensis* under drought stress (**Table 3**), suggesting that their combination mainly promoted the accumulation of AsA by increasing the synthesis of d-glucuronate instead of l-galactose-1P. In addition, with G5 application, the content of d-glucuronolactone was decreased, as shown in the metabolomic analysis of *G. uralensis* under drought stress, which might be due to the downregulation of the gene encoding GME. However, G5 promoted the accumulation of AsA in *G. uralensis* under drought stress, which might be due to the improved production of d-glucuronate with the upregulation of three genes encoding UDPase, two genes encoding GlcAK, and the gene encoding MIOX. On the contrary, Si had no significant effect on AsA content in *G. uralensis* under drought stress, which could be ascribed to the combined effects of the expression of the genes encoding the key enzymes GlcAK, MIOX, ALDH, GGP, APX, and AO.

Regarding GSH metabolism, previous studies have demonstrated that the enzyme GST utilizes GSH and has great potential in plant defense metabolism. Its mechanism helps to reduce the secondary noxious products caused by exposure to stress-induced ROS (Anderson and Davis, 2004). Additionally, the overexpression of GST improved drought tolerance in transgenic *Arabidopsis thaliana* (Xu et al., 2015). In this study, drought stress affected the scavenging ability of ROS in *G. uralensis* through the downregulation of six genes encoding GST. Interestingly, the combination of G5 and Si upregulated three genes but downregulated two genes encoding GST in *G. uralensis* under drought stress, indicating that their combination might reduce the secondary injury products of ROS in *G. uralensis* under drought stress by activating the expression of the GST genes. Among the factors regulating GSH accumulation, GGT catalyzes the transfer of a γ-glutamyl moiety of GSH, GSSG, and other c-glutamyl compounds to amino acids, small peptides, or water (Li et al., 2012; Gu et al., 2020). The transfer of a γ-glutamyl moiety is an important first step in GSH metabolism and recycling and is an essential factor in maintaining redox homeostasis in the cellular environment (Balakrishna and Prabhune., 2014). In the present study, the combination of G5 and Si upregulated the gene encoding GGT and increased the l-glutamate content in *G. uralensis* under drought stress (**Table 2**), which might be due to the promotion of GSH regeneration or proline synthesis from the substrate level (Jones et al., 2021), finally mitigating the cell damage in *G. uralensis* under drought stress.

Furthermore, the AsA–GSH cycle enzymes APX, GPX, AO, DHAR, MDHAR, and GR work together for the detoxification of ROS (Hamid et al., 2010). Previous studies have reported that drought enhanced the enzymatic activities of APX, DHAR, and GR in *Cajanus cajan* (Sreeharsha et al., 2019), but decreased the contents of AsA and GSH and the activities of MDHAR, DHAR, and GR in *Brassica napus* L. (Hasanuzzaman et al., 2018). Herein, drought stress decreased the AsA and GSH levels in *G. uralensis*. This may be the result of the increased APX activity that accelerated the reduction of AsA to monodehydroascorbic acid and the DHAR activity that is related to GSH regeneration, which could be attributed to the upregulation of two genes encoding APX and one gene encoding DHAR. Moreover, the decreased GSH content was attributed to the decreased GPX and GR activities and the downregulated GST genes (**Figure 3**). These results indicate that drought stress reduced the detoxifying co-substrate levels of enzymes such as PODs and GST, thus repressing the ROS scavenging capacity in *G. uralensis.* PGPB and/or Si could, by regulating the AsA–GSH cycle, alleviate the oxidative damage caused by drought stress (Fialová et al., 2018). Under drought stress, PGPB significantly enhanced the activities of APX and GPX in *Ocimum basilicum* L. (Kaushal and Wani, 2016), while Si upregulated the GR activity and increased the AsA content in wheat plants (Gong et al., 2005; Ma et al., 2016). In this study, G5 increased the AsA level by promoting d-glucuronate synthesis and the GSH level by increasing the GR activity, while Si increased the GSH content mainly by increasing the GR activity but decreasing the DHAR activity in *G. uralensis* under drought stress. Thus, the role of G5 or Si in alleviating oxidative stress was related to the increased AsA and GSH contents regulated by the activities of the enzymes related to the AsA–GSH cycle in *G. uralensis* under drought stress, with the specific type of substance and enzyme being different depending on the application of G5 or Si. Notably, G5 combined with Si upregulated three genes encoding GPX, as well as downregulated one gene encoding DHAR, which is consistent with the trend of enzyme activity change at the physiological level. Firstly, G5 combined with Si promoted AsA accumulation by increasing the synthesis of d-glucuronate and accelerated AsA conversion by upregulating the AO gene, enhancing the APX activity, and reducing the DHAR activity, thus providing more substrates for GSH synthesis. The combination of G5 and Si also further boosted the generation of GSH by increasing the GR and GPX activities and upregulating the GST and GGT genes. These results reflect that the combination of G5 and Si enhanced the entire AsA–GSH cycle to maintain its redox balance, which further eliminated the excess ROS that was not removed in time by SOD and CAT, finally resulting in reduced oxidative stress in *G. uralensis* exposed to drought stress; correspondingly, a reduction in plasma membrane permeability was observed.

### G5 and/or Si triggered carotenoid biosynthesis in drought-stressed G*. uralensis* seedlings

As the second line of defense against ROS, carotenoids, comprising some of the important non-enzymatic antioxidants, (Gowayed et al., 2017), play a key role in maintaining cell homeostasis and the antioxidant response in plants (Cicevan et al., 2016). Generally, carotenoids can prevent oxidative damage by scavenging ^1^O_2_, stimulating chlorophyll (Chl*), quenching the triplet sensitizer (3Chl*), dissipating excess energy, and shielding the photosynthetic apparatus (Uarrota et al., 2018). Previous studies reported that the carotenoid contents were reduced in *B. napus*, *Melissa officinalis*, and *Daucus carota* under a water deficit condition (Bukhari et al., 2021; Eshaghi et al., 2022; Zhang et al., 2021b). In the present study, the total carotenoid content was significantly decreased in *G. uralensis* under drought stress (**Figure 4A**), which implied that drought could damage the photosynthetic system and promote carotenoid degradation. Interestingly, PGPB or Si acts as a modulator activating the accumulation of carotenoids in plants, thereby limiting the oxidative injury in various plants caused by drought stress (Yasmin et al., 2017; Khan et al., 2020b; Özdemir, 2021). In this study, G5 and/or Si remarkably increased the total carotenoid content in *G. uralensis* under drought stress, and this increased effect was greater in the combination treatment with G5 and Si than that in G5 or Si treatment alone (**Figure 4A**). It was illustrated that G5 and/or Si might prevent photosystem II overexcitation and regulate the energy dissipation and ROS homeostasis by promoting the accumulation of carotenoids, thus alleviating the oxidative stress caused by drought in *G. uralensis* (Uarrota et al., 2018).

Carotenoids are classified into carotenes and xanthophylls, which mainly include lycopene, α-carotene, β-carotene, lutein, zeaxanthin, violaxanthin, and neoxanthin. Regarding their capacity to scavenge ROS, lycopene, β-carotene, and zeaxanthin can directly reduce oxidative damage in plants (MacDonald et al., 2009; Moise et al., 2014). In the present study, Si or the combination of G5 and Si downregulated the expression of the gene encoding PSY, while the combination of G5 and Si upregulated the expression of the gene encoding Z-ISO in *G. uralensis* under drought stress (**Figures 4B, C** and **Table 4**), indicating that Si alone or its combination with G5 might inhibit phytoene synthesis at the substrate level by inhibiting the PSY activity resulting from the downregulation of the gene; however, the combination of G5 and Si promoted lycopene synthesis through upregulating the expression of the gene encoding Z-ISO, thus preserving the level of lycopene in *G. uralensis* under drought stress (Maass et al., 2009; Efremov et al., 2021). Moreover, the accumulation of lycopene induced by the combination of G5 and Si might further promote the synthesis of β-carotene in *G. uralensis* under drought stress because, apart from the ability to directly scavenge ^1^O_2_, lycopene is a mainly biochemical precursor for the synthesis of α-carotene and β-carotene (Klunklin and Savage, 2017). In addition, the components of the xanthophyll cycle pool play a function in photoprotection and excess energy dissipation, thereby protecting membranes from oxidative damage (Li et al., 2021a). Herein, Si or the combination of G5 and Si downregulated the expression of the gene encoding CHXB and ZEP that is closely related to β-carotene conversion to xanthophylls (**Figures 4B, C** and **Table 4**), illustrating that Si alone or its combined application with G5 might depress the transformation of β-carotene to zeaxanthin and violaxanthin in *G. uralensis*. As a result, the combination of G5 and Si might play a specific role in photosynthetic protection and energy dissipation in drought-stressed *G. uralensis* mainly by promoting the accumulation of lycopene and β-carotene instead of xanthophyll, which coordinated with SOD, CAT, and the AsA–GSH cycle to reduce the oxidative damage in *G. uralensis* induced by drought stress.

### G5 and/or Si activated flavonoid biosynthesis in drought-stressed G*. uralensis* seedlings

Flavonoids, as important plant secondary metabolites that protect plants from drought conditions, are powerful ROS scavengers (Farooq et al., 2021). Generally, they can prevent the generation of ROS in plants through chelating metal ions and inhibiting ROS-producing enzymes such as xanthine oxidase and lipoxygenase (Ferdinando et al., 2012; Sarker and Oba, 2018b), thereby helping to maintain ROS homeostasis and membrane stability and reducing oxidative damage (Ozfidan-Konakci et al., 2020). The flavonoid content was increased by drought stress in wheat (Ma et al., 2014b), but was decreased in *Glycyrrhiza glabra* and *G. uralensis* (Xie et al., 2019; Khaitov et al., 2021). Similarly, drought stress also significantly decreased the total flavonoid content in *G. uralensis* seedlings in the present study, suggesting that the change in the trend of flavonoid content vary according to the plant species or the stress level. Moreover, PGPB inoculation or Si application could reduce the oxidative damage to plants caused by abiotic stresses by stimulating the accumulation of flavonoids (Ma et al., 2016; Chattha et al., 2017). In the present study, G5 or the combination of G5 and Si remarkably increased the total flavonoid content in *G. uralensis* seedlings under drought stress, and this increased effect was greater in the combined G5 and Si treatment than in the G5 treatment alone (**Figure 5A**). These findings suggest that G5 alone or its combination with Si can prevent ROS generation by improving flavonoid accumulation, which could be attributed to the chelation of metal ions and the inhibition of ROS-producing enzymes. Further studies are needed to verify the reasons.

Flavonoid biosynthesis plays a key role in the development of plant tolerance to drought stress (Hahlbrock and Scheel, 1989; Giordano et al., 2016). In this study, the transcriptomic analysis (**Figures 5B, C** and **Table 5**) revealed that drought downregulated the expression of the PAL and HCT genes, but upregulated the expression of the HCT gene in *G. uralensis*, which might have inhibited the transformation of phenylalanine into the key intermediates of flavonoid synthesis in *G. uralensis*. Interestingly, the application of G5 in drought-stressed *G. uralensis* upregulated the gene encoding PAL, but downregulated the genes encoding 4CL and HCT; conversely, Si application upregulated the genes encoding PAL and HCT. These results suggest that G5 might help in the accumulation of cinnamic acid and *p*-coumaroyl-CoA, while Si might enhance the synthesis of caffeoyl-CoA, the downstream metabolite of *p*-coumaroyl-CoA, in drought-stressed *G. uralensis*.

Previous studies have revealed that the genes encoding PAL, HCT, and C4H play important roles in modulating flavonoid biosynthesis in the resistance to oxidative stress in plants (Khakdan et al., 2018). In the present study, the combination of G5 and Si upregulated the genes associated with the synthesis of *p*-coumaroyl-CoA, including PAL, 4CL, and C4H, but downregulated the genes involved in the conversion of *p*-coumaroyl-CoA, such as HCT, CYP98A2, and CCOMT, in drought-stressed *G. uralensis*, indicating that this combination treatment could affect the accumulation of *p*-coumaroyl-CoA by regulating the genes related to its synthesis and transformation, correspondingly affecting the carbon flux distribution and finally enhancing flavonoid biosynthesis in drought-stressed *G. uralensis* (Gallego-Giraldo et al., 2014; Yang et al., 2021).

*p*-Coumaroyl-CoA, a key intermediate in the flavonoid biosynthesis pathway, provides the C6–C3–C6 carbon skeleton to the flavonoid compounds (Yu et al., 2021). After the production of *p*-coumaroyl-CoA, the biosynthetic pathway of flavonoids begins to branch under the activity of CHS, CHR, and CHI (Wang et al., 2015). In the present study, under drought stress, the genes encoding CHS and CHI were downregulated in *G. uralensis*, which could inhibit the activities of CHS and CHI and further inhibit the conversion of *p*-coumaroyl-CoA to its downstream metabolites. Interestingly, in drought-stressed *G. uralensis*, the genes encoding CHS, CHR, and CHI were upregulated by G5 or Si alone, while those encoding CHS and CHR were upregulated by the combination of G5 and Si. These results indicate that G5 and/or Si might accelerate the synthesis of naringenin and liquiritigenin by upregulating the CHS and CHR genes, thus providing sufficient substrate for subsequent antioxidant derivatives (Kim et al., 2004).

As the downstream metabolite of naringenin, kaempferol and quercetin have a significant influence on the scavenging ROS and the detoxification of free radicals, which strengthen tolerance to environmental changes (Tattini et al., 2005; Belinha et al., 2007). In the present study, the expression of the genes encoding F3′,5′H and CYP75B were downregulated in *G. uralensis* by drought stress, which might affect the conversion of kaempferol into quercetin (Wu et al., 2022b). However, the content of 3-*O*-methylquercetin, a derivative of quercetin, was enhanced by G5 application. The expression of the genes encoding F3′,5′H and CYP75B were upregulated by Si, which might accelerate the synthesis of quercetin in *G. uralensis*. These results indicate that G5 or Si might alleviate oxidative stress by increasing the contents of 3-*O*-methylquercetin and quercetin in drought-stressed *G. uralensis*. Notably, in drought-stressed *G. uralensis*, the expression of the genes encoding FLS and CYP75B were downregulated by the combined application of G5 and Si, which could help in the accumulation of kaempferol by inhibiting its degradation, triggering the second stage of the antioxidant system, thus enhancing the tolerance of *G. uralensis* to oxidative stresses induced by drought stress (Ma et al., 2020).

In addition, according to the metabolomic analysis in this study, the downstream metabolites of daidzein, medicarpin, glycitin, and astragalin have different response patterns to drought stress and to G5 and/or Si application (**Figure 5B** and **Table 6**). These flavonoids may be the key to alleviating the oxidative stress caused by drought; however, at present, there are only a few reports on these flavonoids alleviating oxidative stress in plants, and most of the research has been focused on animals (Miriama et al., 2019; Tomar et al., 2020; Wang et al., 2022). Whether they participate in the alleviation of plant oxidative stress needs to be clarified in future research.

## Conclusion

G5 and/or Si have beneficial effects on the antioxidant system of *G. uralensis* seedlings under drought stress, especially their combined application. Through transcriptional and physiological regulation, the combination of G5 and Si increased the activities of SOD and CAT, enhanced the entire AsA–GSH cycle, and triggered the accumulation of the major antioxidant carotenoids and flavonoids in *G. uralensis* exposed to drought stress. This might have contributed to maintaining redox balance by increasing the levels of AsA and GSH and the activities of the key enzymes, including SOD, CAT, APX, GPX, and GR, and upregulating the expression levels of the related genes such as GPX, AO, and GST, in addition to protecting the photosynthetic activity and dissipating excess energy through facilitating the accumulation of total carotenoids and total flavonoids by modulating the key genes related to their biosynthesis processes, thus protecting *G. uralensis* seedlings from membrane lipid peroxidation induced by drought stress. Our findings not only contribute to an in-depth understanding of the role of the combined application of G5 and Si in *G. uralensis* under drought stress based on our previous studies but also provide new insights into the comparative and synergistic roles of PGPB and Si in the antioxidant system of plants exposed to drought conditions.

## Data availability statement

The data presented in the study are deposited in Sequence Read Archive (SRA) database at NCBI (SRA BioProject PRJNA885037).

## Author contributions 

XZ designed the experiment, performed and supervised the project, and edited and reviewed the manuscript. XM wrote the manuscript, analyzed the data, and performed the experiments. ZX, DL, LZ, and WZ performed the experiments. All authors contributed to the article and approved the submitted version.

## Supplementary material

Supplementary Figure Bio-compatibility interaction of G5 and Si. The G5 (200 μL) was uniformly coated on the plate, and a hole with a radius of 0.5 mm was punched using a sterile punch, and 200 μL of Si (2 mM K_2_SiO_3_) was injected into NA plates for bio-compatibility interaction testing at 28°C. Finally, based on the existence or absence of a bacteriostatic band during 24 to 48 hours, it is determined whether the two can be used in combination.

## Glossary

**Table:**

ROS	reactive oxygen species
PGPB	plant growth-promoting bacteria
Si	silicon
G5	*Bacillus pumilus*
SOD	superoxide dismutase
CAT	catalase
POD	peroxidase
AsA	ascorbate
GSH	glutathione
*G. uralensis*	*Glycyrrhiza uralensis* Fisch.
APX	ascorbate peroxidase
GPX	glutathione peroxidase
MDHAR	monodehydroascorbate reductase
DHAR	dehydroascorbate reductase
GR	glutathione reductase
$O_{2}^{\cdot -}$	superoxide
H_2_O_2_	hydrogen peroxide
MDA	malondialdehyde
DHA	dehydroascorbate
GSSG	glutathione disulfide
UGDH	UDP-glucose 6-dehydrogenase
UDPase	UDP-sugar pyrophosphorylase
GlcAK	glucuronokinase
MIOX	inositol oxygenase
ALDH	aldehyde dehydrogenase (NAD^+^)
GGP	GDP-l-galactose phosphorylase
GME	GDP-mannose-3′,5′-epimerase
AO	l-ascorbate oxidase
G6PDH	glucose-6-phosphate 1-dehydrogenase
GGT	gamma-glutamyl transpeptidase
APN	aminopeptidase N
CHXB	β-carotene hydroxylase
PSY	phytoene synthase
Z-ISO	ξ-carotene isomerase
ZEP	zeaxanthin epoxidase
PAL	phenylalanine ammonia lyase
4CL	4-coumarate-CoA ligase
C4H	*trans*-cinnamate 4-monooxygenase
HCT	shikimate *O*-hydroxycinnamoyltransferase
CYP98A2	5-*O*-(4-coumaroyl)-d-quinate 3′-monooxygenase
CCOMT	caffeic acid 3-*O*-methyltransferase/acetylserotonin *O*-methyltransferase
CHS	chalcone synthase
CHI	chalcone isomerase
CHR	chalcone reductase
CYP81E1/E8	isoflavone/4′-methoxyisoflavone 2′-hydroxylase
VR	vestitone reductase
UGT	isoflavone 7-*O*-glucosyltransferase
MAT	isoflavone 7-*O*-glucoside-6"-*O*-malonyltransferase
FLS	flavonol synthase
F3′,5′H	flavonoid 3′,5′-hydroxylase
CYP75B	flavonoid 3′-monooxygenase
CPM	counts per million
VIP	variable importance in projection
