# Supplementary figures and images for: Comprehensive physiological, transcriptomic, and metabolomic analyses reveal the synergistic mechanism of Bacillus pumilus G5 combined with silicon alleviate oxidative stress in drought-stressed Glycyrrhiza uralensis Fisch
Source: Front Plant Sci. 2022 Dec 8;13:1033915. doi: 10.3389/fpls.2022.1033915 (PMC9773211; doi:10.3389/fpls.2022.1033915)

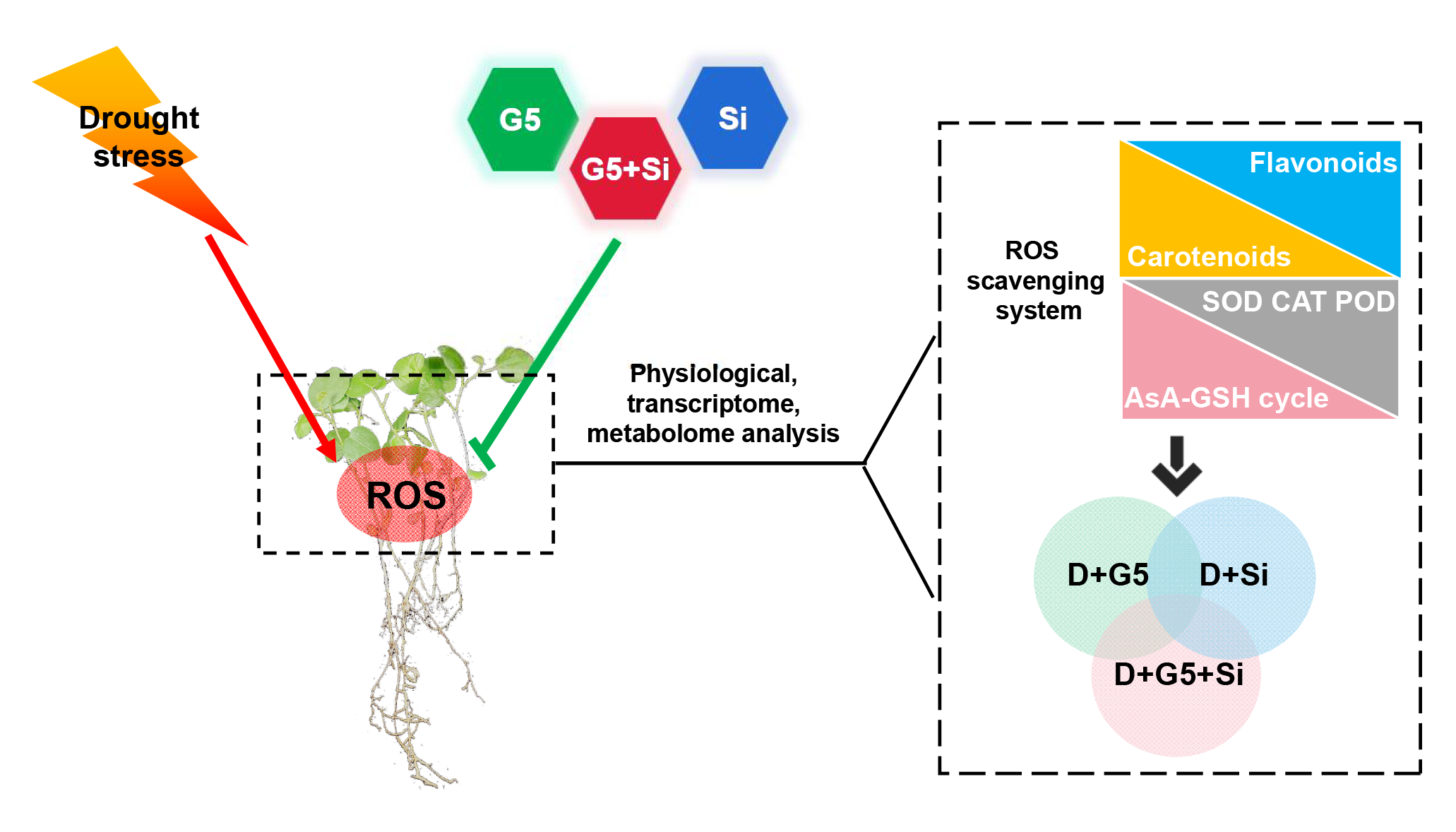

Supplement: Supplementary file 1 [file Image_2.tif]

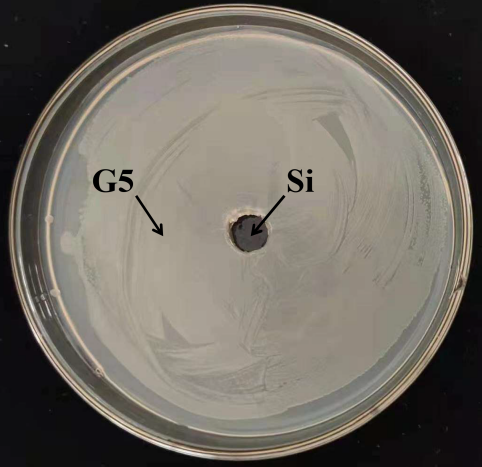

Supplement: Supplementary Figure — Bio-compatibility interaction of G5 and Si. The G5 (200 μL) was uniformly coated on the plate, and a hole with a radius of 0.5 mm was punched using a sterile punch, and 200 μL of Si (2 mM K2SiO3) was injected into NA plates for bio-compatibility interaction testing at 28°C. Finally, based on the existence or absence of a bacteriostatic band during 24 to 48 hours, it is determined whether the two can be used in combination. [file Image_1.tif]
